# Case Report: Posterior Reversible Leukoencephalopathy Syndrome (PRES) as a Biologically Predictable Neurological Association in Severe COVID-19. First Reported Case From Australia and Review of Internationally Published Cases

**DOI:** 10.3389/fneur.2020.600544

**Published:** 2021-01-27

**Authors:** Tissa Wijeratne, Chanith Wijeratne, Leila Karimi, Carmela Sales, Sheila Gillard Crewther

**Affiliations:** ^1^School of Psychology and Public Health, La Trobe University, Melbourne, VIC, Australia; ^2^Department of Neurology, Western Health & University Melbourne, Australian Institute for Musculoskeletal Science (AIMSS), Western Centre for Health Research & Education (WHCRE), Sunshine Hospital, St Albans, VIC, Australia; ^3^Department of Medicine, Faculty of Medicine, University of Rajarata, Anuradhapura, Sri Lanka; ^4^Monash Medical School, Clayton, VIC, Australia

**Keywords:** posterior reversible encephalopathy syndrome (PRES), COVID-19, SARS-CoV-2, hypertension, case report

## Abstract

Reports of different types of neurological manifestations of COVID-19 are rapidly increasing, including changes of posterior reversible leukoencephalopathy syndrome (PRES). Here we describe the first reported case of COVID-19 and PRES in Australia diagnosed on basis of MRI brain imaging and confirmed clinically by presence of confusion, delirium, headaches, also associated with hypertension and blood pressure variability and stable long-term kidney problems. He made full recovery as his blood pressure was controlled and clinical status was supported with appropriate supportive therapy. Although traditionally a rare condition, PRES is likely to be more common among patients with COVID-19 pathobiology there is Renin downregulation of ACE2 receptors, involvement of Renin-Angiotensin-Aldosterone system, endotheliitis, cytokine storm, and hyper-immune response. Thus we advocate clinical suspicion and early brain imaging with MRI brain among vulnerable patients with known co-morbidities, and diagnosed with COVID-19 given that hypertension and blood pressure variability are often exacerbated by acute SARS-CoV-2 immune reactions. Such acute hypertensive encephalopathy was able to be reversed with timely supportive therapy ensuring re-hydration and re-establishment of blood pressure control.

## Introduction

A new β corona virus, severe acute respiratory syndrome coronavirus2 (SARS-CoV2) emerged as a novel cause of pneumonia in December 2019. Since then the SARS-CoV2 has spread to over 216 countries and is now regarded as a major world pandemic (1). As of 21st of December, the mortality rate of COVID-19 (disease caused by SARS-CoV2 infection in humans) is being reported as 2.20% with the number of confirmed deaths as 1,699,878 and 77,184,964 recorded cases worldwide. Reports of different types of neurological manifestations of COVID-19 are rapidly increasing including a number involving COVID19 and posterior reversible leukoencephalopathy syndrome (PRES) and acute disseminated encephalomyelitis (ADEM) (2) confirmed with brain imaging). Several studies have described that Corona viruses are associated with CNS disease such as ADEM (3–5) as evidence of more long-lasting impact.

We present, to our knowledge the first reported case of posterior reversible encephalopathy syndrome (PRES) associated with severe acute respiratory syndrome coronavirus2 (SARS-CoV2) in Australia. A comprehensive literature review revealed that 18 cases since December 2019 had been documented worldwide by late August 2020 though as shown in Table 1 it is noteworthy than only 5/14 cases occurred in COVID patients without serious history of co-morbidities.

**Table 1:** Studies of PRES in COVID-19.

**Author**	**Study design**	**Age/Sex**	**Comorbidities**	**Laboratory features**	**Neuroimaging features**
Coolen et al. (6)	Case series	NA	NA	NA	Superior parietal precentral and parieto- occipital cortico- subcortical swelling with marked supratentorial white matter changes
Franceschi et al. (7)	Case series	48M	Obesity	NA	Vasogenic edema posterior parieto- occipital region, extensive petechiae on SWI throughout the corpus callosum
		67F	HTN, DM, IHD, Gout	NA	Restricted diffusion with edema of the parieto-occipital lobes, right frontal, basal ganglia, and cerebellar hemispheres
Princiotta Cariddi et al. (8)	Case report	64F	HPN, AF, Dyslipidemia, OSA, hyperuricemia	High CRP Normal CSF NLR = 7	Bilateral parieto-occipital FLAIR changes with subacute hemorrhages
Parauda et al. (9)	Case series	64–74 (2M, 2F)	1) HT,DM 2) DM, Dyslipidemia	High D-dimer, ferritin, LDH, CRP	Parieto-occipital FLAIR changes, microbleeds in SWI
Doo et al. (10)	Case series	55M, 64M	1) DM 2) Ex-smoker	NA	Extensive parieto-occipital edema
Kaya et al. (11)	Case report	38M	None	High CRP and ferritin, marked lymphopenia	Extensive edema of bilateral (left occipital, frontal cortical splenium of the corpus callosum)
Gomez-Enjuto et al. (12)	Case report	74M	Multiple myeloma	NA	Bilateral parieto-occipital and frontal FLAIR changes
Conte et al. (13)	Case report	63F	None	NA	FLAIR hyperintensities in both hemispheres, evidence of SAH with effusion on left pre-central sulcus, gad-enhancement in the posterior white matter
Rogg et al. (14)	Case report	59M	None	NA	FLAIR changes in the posterior white matter
Kishfy et al. (15)	Case series	58M 67F	1) HTN, Dyslipidemia 2) HTN, Obesity	High inflammatory markers in both with nadir in recovery phase	FLAIR hyperintensities in both occipital lobes, both temporal lobes
Anand et al. (16)	Case series	61F	None	NA	Symmetric white matter T2 hypertintensities involving the parieto-occipital lobe without diffusion restriction
		62F	HIV	Crea 4.33 mg/dL CSF: high protein, high glucose with pleocytosus	Diffuse white matter T2 hypertintensities involving the parieto-occipital, frontal, and temporal loves with partial sulcal effacement

A 55-year-old man with known hypertension compliant with medications, obesity, chronic renal impairment secondary to hypertensive nephropathy [baseline eGFR 24 (normal >60)], a 35 pack-year history of smoking, obstructive sleep apnea and hypercholesterolaemia was part of a family cluster of acute infections with SARS-CoV2 1 week prior to the admission to our hospital. The index case and the family (wife and four children) were positive for SARS-CoV2. In particular, he was experiencing headaches, fever, and dry cough for the prior 7 days. There was no report of nausea and/or vomiting during the preceding week. Prior to hospitalization, the family physician had prescribed oral dexamethasone 6 mg daily. On day seven, his daughter and wife found to him significantly lethargic, confused and disorientated and brought him to the Emergency Department (E.D) where he was admitted to hospital.

**Figure 1:**
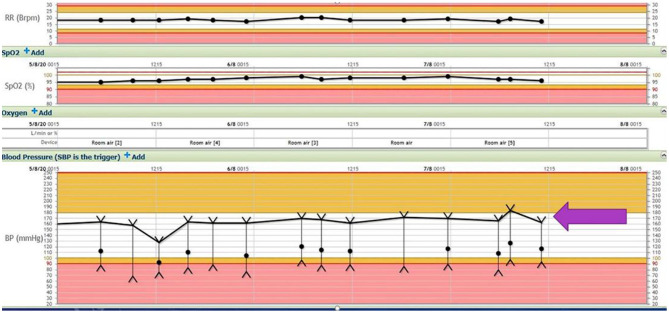
Respiratory rate, oxygen saturation, and blood pressure variability (block arrow) throughout the hospital stay (SBP 180–140 mmHg, DBP 60–100 mmHg).

Apart from his altered mentation, he appeared normal on examination in the ED. At the time of admission, his respiratory rate was 18 and his blood pressure was 171/85 mmHg, with a mean arterial pressure of 116. His blood pressure variability is shown in Figure 1 below.

His blood examination displayed a total white cell count (WCC) of 11.5, a neutrophil count of 9.9 and a lymphocyte count of 0.6. His neutrophil-lymphocyte ratio (NLR) was 16.5 compared to expected value of <2 for his age group. C- reactive protein was also significantly elevated (132 mg/L compared to expected <5 mg/L) at admission. There was mild elevation in his creatinine levels from baseline. He was maintained on Moxonodine 200 mcg bid and his usual blood pressure medication, Prazosin 1 mg bid. He was found to be coherent on the third day after admission and was discharged home. On discharge, his total WCC had improved to 7.8, with a neutrophil count of 5.8 and lymphocyte count of 0.6. His NLR was thus 13.

**Figure 2:**
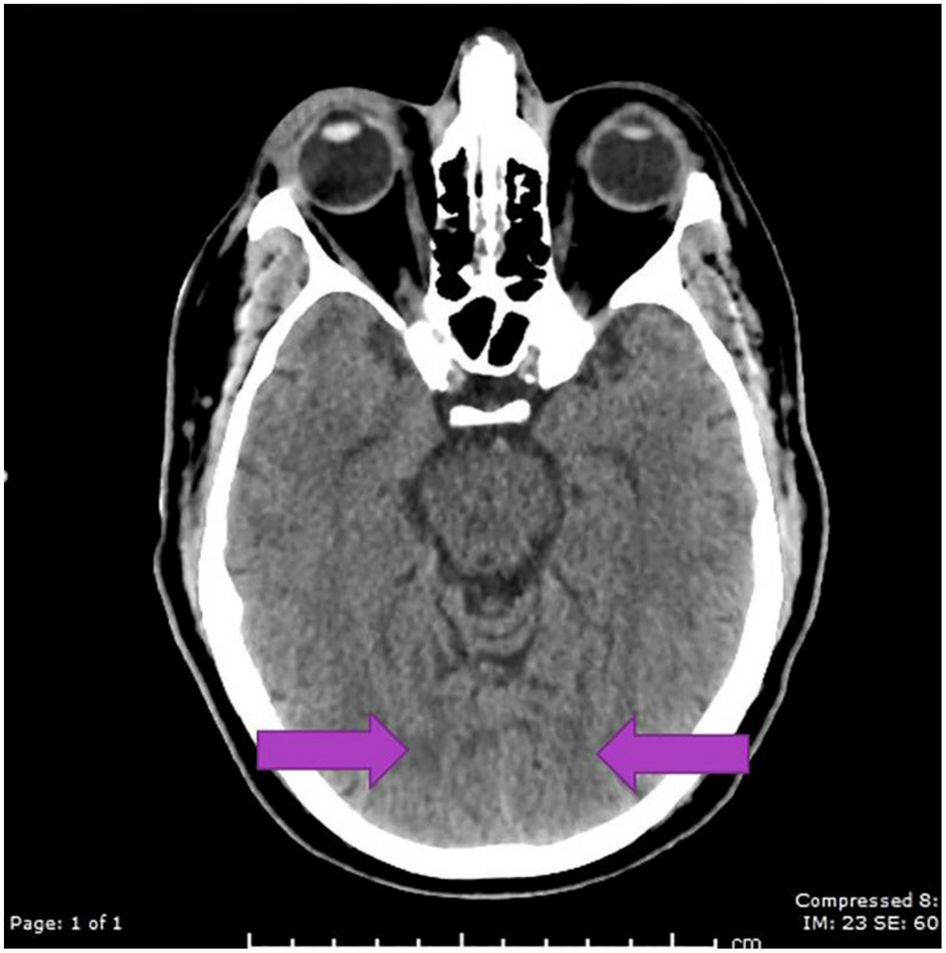
C.T. scan with bilateral posterior parietal and occipital hypo intensities suggestive of PRES.

**Figure 3:**
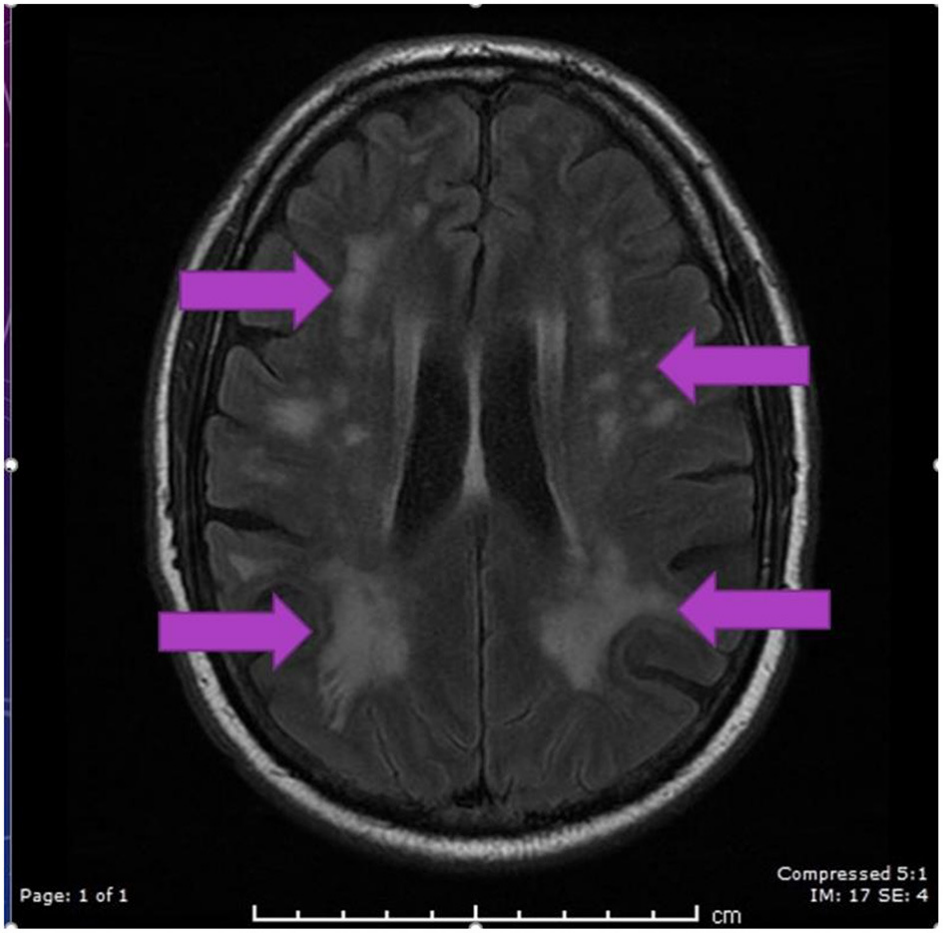
MRI axial FLAIR image demonstrating hyperintensities in periventricular regions in both parietal, occipital and frontal regions secondary to PRES.

**Figure 4:**
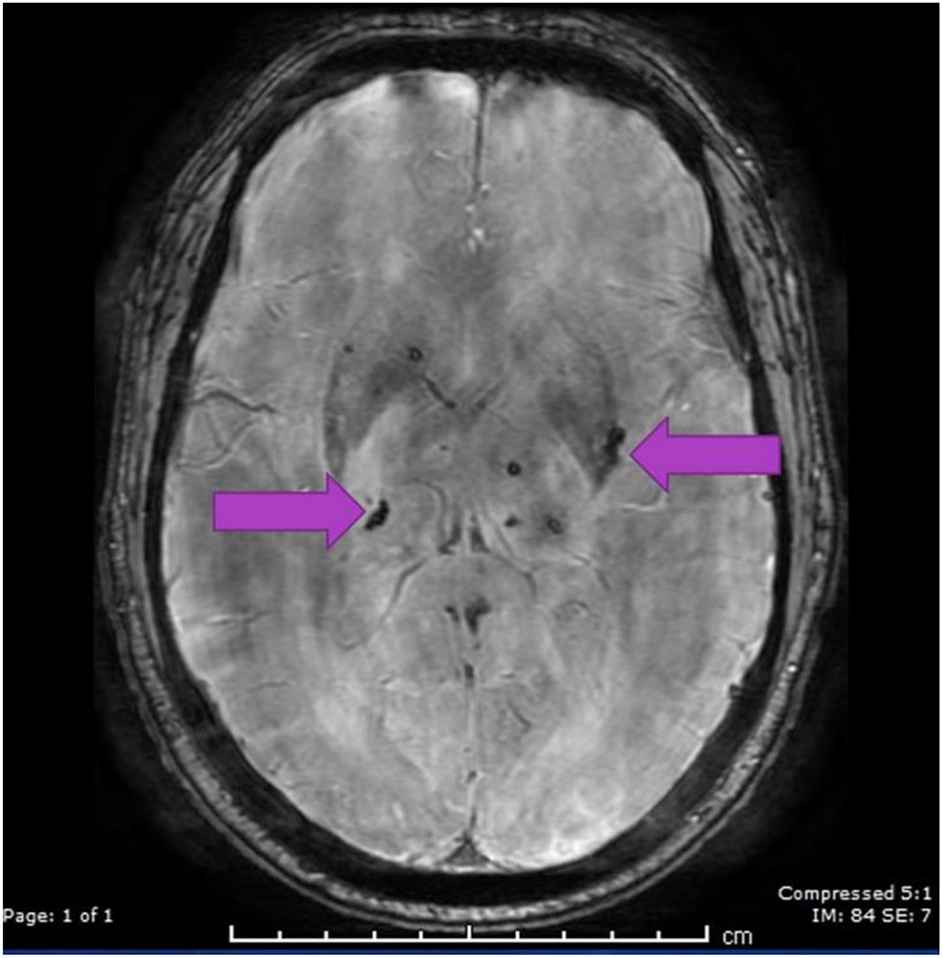
SWI image showing cerebral microbleeds in the basal ganglia region.

A CT scan of his brain on admission showed bilateral hypointensities around his posterior parietal-occipital regions (see Figure 2). A subsequent cranial MRI taken on the same day revealed bilateral parieto-occipital T2 FLAIR (fluid attenuation and inversion recovery) hyperintensities compatible with PRES given the recovering symptoms compared to VANDAL with severe illness. There were diffuse petechial hemorrhages (17) shown on SWI (susceptibility-weighted images) throughout the basal ganglia and deep white matter indicative of cerebral microbleeds (Figures 3 and 4). Multiple small foci of increased diffusion weighted imaging (DWI) signals with corresponding low apparent diffusion coefficient (ADC) signal were also noted in the deep white matter of the bilateral centrum semi ovale and corona radiata (not shown as the changes are barely visible on the workstation console even). These could potentially be related to chronic hypertension, although possibilities involving acute COVID-19 related microangiopathy cannot be completely discounted [well-described in VANDAL (18)].

## Discussion

Almost 25 years have elapsed since PRES was first described (19). Interestingly, the COVID19 pandemic has also shown a marked increase in the number of PRES cases as indicated in Table 1. Readily available MRI brain imaging now helps clinicians diagnose these conditions (PRES as well as VANDAL) easily though imaging after COVID infection is unlikely to be definitive if prior scans are not available. Despite the poor understanding of the exact pathophysiology of PRES, several different potential pathogenic mechanisms have been suggested. These include endothelial injury related to rapid changes in blood pressure (particularly hypertension) and the effects of infections like SARS-CoV-2 and cytokines on the endothelium disrupting the blood-brain barrier and causing associated cerebral oedema and changes to the auto-regulation of intracranial pressure raising the possibility of VANDAL, in milder form.

Normal clinical presentation of PRES includes altered mentation, headaches, visual disturbances, and seizures in patients with other underlying comorbidities. Fluctuations in blood pressure are a characteristic sign of PRES both in COVID patients and in other non-COVID associated diagnosis.

A leading theory of the pathophysiology of PRES suggests that rising hypertension exceeding the upper limit of autoregulation of cerebral blood flow culminates in hyperfusion and blood- brain barrier disruption along with the extravasation of macromolecules and plasma to interstitial tissues (20, 21).

Acute hypertension has been suggested to cause endothelial dysfunction in susceptible patients as multiple mechanisms lead to an eventual breakdown of the blood-brain barrier (22). The patient's mean baseline blood pressure, blood pressure variability, proportional rise in blood pressure and rapidity of changes to blood pressure are all key factors that could potentially lead to blood-brain barrier breakdown and thus vasogenic edema (21). The direct effects of excessive circulating cytokines can cause endothelial dysfunction and PRES (23). The relationship between COVID-19 and endothelial dysfunction is notably well-recognized (24, 25).

Furthermore, it is known that COVID 19 attaches to ACE 2 receptors on endothelial cells (lung parenchyma as well as brain parenchyma) and brain microglia (26) inducing an alteration in the Renin Angiotensin Aldosterone System (RAAS) that favors the classical pathway, resulting in vasoconstriction and potential changes in blood pressure (27). This may directly or indirectly affect the cerebral vasculature, which may lead to PRES (20) or the recently described Viral Associated Necrotizing Disseminated Acute Leukocepthalopathy (18, 28).

There is no specific treatment for PRES, but symptoms are thought to be reversible once the underlying cause is removed (21). It is widely believed that appropriate treatment of hypertension, and associated inflammation is of great importance for treating PRES. A main theory of the pathophysiology of PRES suggests that rapidly rising hypertension particularly in patients with kidney problems overshoots the upper limit of autoregulation so that insufficient cerebral autoregulation leads to ongoing hyper perfusion, disruption of the blood brain barrier, endothelial dysfunction, and oedema (21). This theory is supported by a series of papers suggesting the existence of a relationship between acute hypertension and PRES relationship as well as showing that clinical and radiological improvement may be brought about by appropriate treatment of blood pressure.

While the pathophysiology of PRES or COVID induced VANDAL symptoms remains controversial, we advocate for tight blood pressure control and MRI imaging in all COVID-19 patients who show neurological symptoms from headaches to confusion and cognitive impairment, particularly in those with hypertension, as a matter of priority both at the time of the admission as well as during recovery prior to leaving hospital. We could not perform the repeat brain imaging due to the rapid recovery of the patient and difficult access to imaging in the middle of the pandemic related logistic issues. Uncontrolled hypertension and blood pressure variability alongside viral inflammation and excessive immune responses are both potential but not unexpected risk factors for worse outcomes involving COVID-19 and its effects on vulnerable kidney patients, given its propensity to attack the RAAS system and ACE2 receptors. We hypothesize that hypertension and potentially blood pressure variability exacerbated by acute SARS-CoV-2 action on the RAAS and associated inflammation may be additional risk factors for endothelial dysfunction and hypertensive encephalopathy with modest blood pressure changes during acute infection with SARS-CoV2 (20, 21, 29–32).

While most patients will fully recover from PRES, the extent of recovery and exactly how reversible symptoms of PRES or VANDAL are, is not known, given the definitions of both disorders are of a symptom of an underlying co-morbidity and particularly now in the presence of COVID. In a postmortem brain MRI study Coolen et al. (6), described hemorrhagic and PRES related brain lesions in non-survivors of COVID-19 (3). Hemorrhagic lesions are also not uncommon among non-COVID related PRES, but with patterns such as intraparenchymal and subarachnoid hemorrhage favoring the later (24, 25). These premises also make PRES a misnomer in several ways (2). Interestingly, Agarwal et al. also described eight cases of critically ill COVID19 cases who demonstrated diffuse changes in the white matter evolving into necrotizing cystic cavitation after a few weeks, an entity which has been termed as Virus- Associated Necrotizing Disseminated Leukoencephalopathy (VANDAL) (26). In contrast to the outcomes described in this series, the index patient described had a good prognosis. Whether an overlap between COVID-related PRES and VANDAL exists need further investigation though is likely.

More research needs to be done into the specifics of both PRES and VANDAL disorders. COVID-19 is a very likely risk factor for PRES except the oedema seen is often more than that induced preferentially by the basilar artery. Thus, we argue that more vigilance is required to detect cognitive and neurological symptoms in these patients and to facilitate appropriate neuroimaging earlier enough and then again when symptoms dissipate. Surveillance neuroimaging is also necessary to determine radiologic outcomes. Tight control of blood pressure and reducing the risk of blood pressure variability and inflammation and hence potential for any further cytokine storms that are likely initiators endothelial cell damage allowing fluid leakage to the brain are likely to be helpful in this context.

## Data Availability Statement

The raw data supporting the conclusions of this article will be made available by the authors, without undue reservation.

## Ethics Statement

Ethical review and approval were not required for the study on human participants in accordance with the local legislation and institutional requirements. The patients/participants provided their written informed consent to participate in this study. Written, informed consent was obtained from the participant for the publication of this case report (including all data and images).

## Author Contributions

TW conceived the idea and wrote the first draft of the manuscript. CW, CS, LK, and SC edited and contributed to the final manuscript. All authors approved the final manuscript.

## Conflict of Interest

The authors declare that the research was conducted in the absence of any commercial or financial relationships that could be construed as a potential conflict of interest.
